# Supplementary material for: Conditional chemoconnectomics (cCCTomics) as a strategy for efficient and conditional targeting of chemical transmission
Source: eLife. 2024 Apr 30;12:RP91927. doi: 10.7554/eLife.91927 (PMC11060718; doi:10.7554/eLife.91927)
Supplement: Figure 3—source data 3. [file elife-91927-fig3-data3.zip › Figure 3 source data3 part 2.zip!/Figure 3-Source data 3-part2/1062-13-CRISPResso_Report_jt28fhr9.zip!/CRISPResso_on_1062_14/1c.Alignment_barplot.pdf]

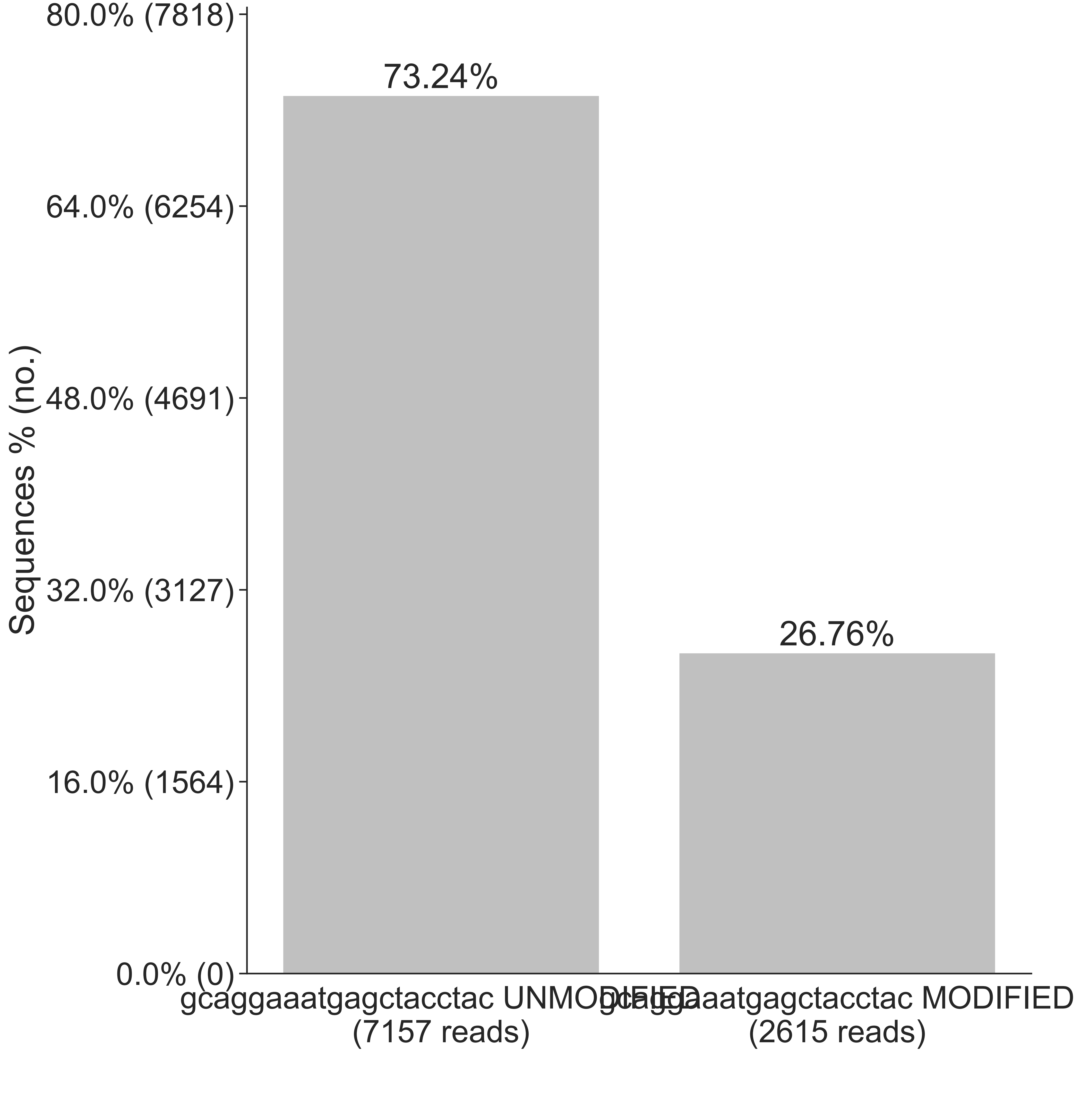

80.0% (7818)
64.0% (6254)
48.0% (4691)
32.0% (3127)
16.0% (1564)
0.0% (0)
Sequences % (no.)
73.24%
26.76%
gcaggaaatgagctacctac UNMODIFIED
(7157 reads)
gcaggaaatgagctacctac MODIFIED
(2615 reads)
